# Supplementary material for: Z-Scheme Heterojunction of Phosphorus-Doped Carbon Nitride/Titanium Dioxide: Photocatalytic Performance
Source: Molecules. 2024 Sep 12;29(18):4342. doi: 10.3390/molecules29184342 (PMC11433829; doi:10.3390/molecules29184342)
Supplement: Supplementary file 1 [file molecules-29-04342-s001.zip › molecules-3195426-supplementary.pdf]

Supporting Information

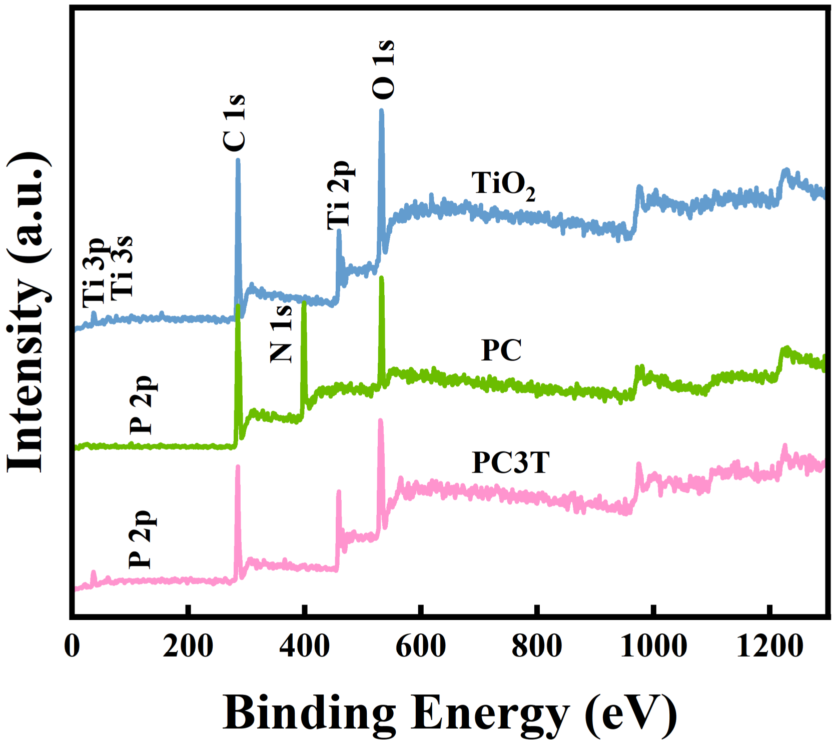

Figure S1 XPS spectra of full spectrum of TiO_2, PC, and PC3T.

Table S1: Surface areas and pore sizes of all the samples.

Sample	Surface Area (m^2/g)	Pore Width (nm)
TiO_2	35.43	8.17
PC	9.13	1.95
PC1T	12.22	1.66
PC2T	16.43	1.58
PC3T	21.34	14.87
PC4T	20.15	1.63
